# Retraction Note: Preoperative versus postoperative ultrasound-guided rectus sheath block for improving pain, sleep quality and cytokine levels in patients with open midline incisions undergoing transabdominal gynecological surgery: a randomized controlled trial

**DOI:** 10.1186/s12871-021-01437-z

**Published:** 2021-09-23

**Authors:** Feng Jin, Zhe Li, Wen-fei Tan, Hong Ma, Xiao-qian Li, Huang-wei Lu

**Affiliations:** Department of Anesthesiology, the First Hospital of China Medical University, Shenyang, 110001 China

**Retraction Note: BMC Anesthesiol 18, 19 (2018)**

**https://doi.org/10.1186/s12871-018-0485-9**

The Editor has retracted this article due to concerns about the data. Part of Fig. 2B is identical to part of Fig. 2A in a different paper by some of the same authors [1]. It was found that the raw data used to generate the figures was identical between two time-points. The authors have subsequently informed the journal that the incorrect data was used to generate the figure in this article. As the data on which the findings are based are not reliable, the article has been retracted.

Author Wen-fei Tan and Feng Jin agree to this retraction. Author Zhe Li, Hong Ma, Xiao-qian Li and Huang-wei Lu have not responded to any correspondence from the Editor about this retraction.
